# Deep Learning Based Pavement Inspection Using Self-Reconfigurable Robot

**DOI:** 10.3390/s21082595

**Published:** 2021-04-07

**Authors:** Balakrishnan Ramalingam, Abdullah Aamir Hayat, Mohan Rajesh Elara, Braulio Félix Gómez, Lim Yi, Thejus Pathmakumar, Madan Mohan Rayguru, Selvasundari Subramanian

**Affiliations:** 1 Engineering Product Development Pillar, Singapore University of Technology and Design (SUTD), Singapore 487372, Singapore; abdullahaamir@sutd.edu.sg (A.A.H.); rajeshelara@sutd.edu.sg (M.R.E.); resor95@gmail.com (B.F.G.); yi_lim@mymail.sutd.edu.sg (L.Y.); pathmakumar_thejus@mymail.sutd.edu.sg (T.P.); mmrayguru87@gmail.com (M.M.R.); 2 Layorz Private Limited, Tamil Nadu, Karur 639117, India; selvasundari@layorz.com

**Keywords:** pavement cracks detection, garbage detection, machine learning, self-reconfigurable, pavement sweeping robot

## Abstract

The pavement inspection task, which mainly includes crack and garbage detection, is essential and carried out frequently. The human-based or dedicated system approach for inspection can be easily carried out by integrating with the pavement sweeping machines. This work proposes a deep learning-based pavement inspection framework for self-reconfigurable robot named Panthera. Semantic segmentation framework SegNet was adopted to segment the pavement region from other objects. Deep Convolutional Neural Network (DCNN) based object detection is used to detect and localize pavement defects and garbage. Furthermore, Mobile Mapping System (MMS) was adopted for the geotagging of the defects. The proposed system was implemented and tested with the Panthera robot having NVIDIA GPU cards. The experimental results showed that the proposed technique identifies the pavement defects and litters or garbage detection with high accuracy. The experimental results on the crack and garbage detection are presented. It is found that the proposed technique is suitable for deployment in real-time for garbage detection and, eventually, sweeping or cleaning tasks.

## 1. Introduction

The development of urban pavement infrastructure systems is an integral part of modern city expansion processes. Every year, the pavement infrastructure has been growing multiple folds due to developing new communities and sustainable transport initiatives. Maintaining a defects free, clean, and hygienic pavement environment is a vital yet formidable Pavement Management System (PMS) task. Pavement inspection, i.e., identifying defects and litter or garbage with cleaning, are mandatory to achieve a defects-free and hygienic pavement environment. Generally, in PMS, human inspectors are widely used for defect and cleanness inspection. However, this method takes a long inspection time and needs a qualified expert to systematically record the severity of defects and mark defects’ spatial location. Furthermore, routine cleaning of lengthy pavement is a tedious task for sanitary workers.

Autonomous robots are suited for repetitive, dull, dirty, tedious, and time-consuming tasks. The emphasis on automation in construction using robots is reported in [1] where a detailed study on how robots potentially value add to the construction workflow, quality of work and project timeline in less explored areas of construction robotics. Tan et al. [2] introduced robot inclusive framework targeting robots for construction sites, that proposes a measure of robot-inclusiveness, different categories for robot interaction, design criteria and guidelines to improve robot interaction with the environment.

An initial attempt for autonomous sweeping using a robotic system was reported in [3], where the robot is designed to autonomously sweep road curbside as current methods to clean road curbside is very labour intensive and repetitive. Pavement cleaning robots have many design limitations, such as the robots are of fixed shape and cannot cover the different sizes of pavement width, and are not equipped with real-time garbage and pavement crack detection algorithms. As a result, limited efficiency is achieved during the pavement cleaning tasks.

### 1.1. Literature Review

Self-reconfigurable robots are becoming a viable alternative for fixed morphology robots. These robots are developed with an inherent capability to autonomously change their kinematics [4] to overcome difficulties in handling a given task and traversing the environment. The advantage of using a self-reconfigurable robot named hTetro over fixed-shaped robots for indoor cleaning is demonstrated in [5,6]. The self-reconfigurable robot application was extended for an outdoor pavement sweeping robot named Panthera with its design disclosed in [7] and its vision-based reconfiguration ability based on pedestrian detection and their velocity was demonstrated in [8,9]. The Panthera robot’s autonomy index is reported as 2.4 on the scale of 10 using the framework reported in [10]. Panthera’s previous work does not include the garbage and pavement inspection task, which is an essential aspect. This paper aims to increase Panthera robot’s use case and index by autonomous inspection of pavement and its geotagging information. Also, the crack detection scheme will be useful for extending it to the drain inspection robots as reported in [11].

Computer vision with Machine Learning (ML) and Deep Learning (DL) based defect, and cleanness inspection is an emerging technique [12,13,14,15,16,17,18]. It has been widely used for the detection of material defects, drivable region detection in autonomous vehicle, waste management industries [19,20,21]. In contrast with manual inspection scheme, computer vision with ML-based inspection methods are faster, high-precision, and more suitable for routine infrastructure and cleanness inspection task. Emanuel et al. [22] using image percolation to detect cracks and demonstrated that it is robust to blurring or image quality degradation. An autonomous crack inspection robot has been implemented in [23] where it is able to process the image data fast, with low cost and in variable lighting conditions. Fan et al. [24] proposed an enhanced road crack detection scheme using the Deep Convolution Neural Network (DCNN), bilateral filtering, and adaptive threshold algorithms. Here, DCNN was used to determine the defect in the image, bilateral filtering for smoothed the crack region, and adaptive threshold method from extract the cracks from the road surface. The real-time road crack mapping system was proposed in [25] where the crack detection network was trained with longitudinal, transverse, and alligator type defects images and optimized by the Bayesian optimization algorithm. The author reported that the crack detection network classifies the road defects with 97% accuracy. The deep neural network system for the detection of cracks on road were demonstrated in [26]. Here, the pavement inspection which includes the detection of garbage apart from the cracks and potholes in road conditions is carried out.

The asphalt pavement crack detection and classification system were reported in [27]. The detection network in [27] was built with three convolution pooling layers and two fully connected layers. The trained model obtained was having an accuracy of 98% defect detection. In [28] Ting yang et al proposed modified SegNet based scalable crack detection model for inspecting concrete and asphalt pavement and bridge deck cracks. The CNN network was built with VGG16 net without the top layer, initialized with open-source pre-trained weights, trained with 2000 high-resolution crack images, and achieve 83% defect detection accuracy. In another study [29], YOLOV2 deep learning framework was trained to automated pavement distress analysis. The network was trained with 7240 images, and the trained model obtained an F1 score of 0.8780 for distress detection. Besides, the author reported that the network accurately detects the alligator cracks but struggles with transverse cracks. Another study reported on Sobel and Canny edge detection approach for detecting pavement cracks and attain an Classification Accuracy Rate (CAR) as 79.99% reported in [30]. However, most of the defect inspection scheme was used offline, and very few works have reported on the pavement cleanness inspection using a deep learning scheme.

The CNN based approaches were reported in the literature for its effectiveness in recognizing garbage, cleanness inspection, and garbage sorting. Chen et al. proposed a computer vision-based robot grasping system for automatically sorting garbage. Here, Fast Region Convolution Neural Network (F-RCNN) is employed for detecting different objects in a given scene [31]. Gaurav et al. [32] developed a smartphone application called SpotGarbage to detect and locate debris outdoors. A pre-trained AlexNet CNN model was used to detect the garbage in images captured outdoors. The training images were obtained using Bing’s image search API. The model has achieved a classification accuracy of 87% for this application. However, it only reports on the garbage detected or not detected as a heap, while not considered the type of objects in the garbage which is also the focus of present work in context of pavements.

An alternative approach that involves the use of a Support Vector Machine (SVM) with Scale Invariant Feature Transform (SIFT) functionality to identify the recyclable waste is provided in [33]. Images of solid waste are used in this method for classification, but it fails to identify the location of garbage. However, 94% of accuracy is achieved for the given task. Rad et al. [34] has proposed a model using overfeat-googlenet to outdoor garbage detection. In this work, 18,672 images of various types of garbage are used to train a Convoluted Neural Network (CNN) in the identification of solid waste from outdoor environments, such as newspapers, food containers, cans, etc. The network reached an accuracy of 68.27% for the detection of debris in this application. Similar work was carried out for for identification and classification of solid and liquid debris using the MobileNet V2 Single Shot Detector (SSD) framework and the SVM model was used to estimate the size of liquid spillage [35]. Recently, Fulton et al. [36] have proposed a deep-learning framework based debris detector for underwater vehicles. As an outcome of their study, CNN and SSD have better performance metrics when compared with YOLOV2 and Tiny-YOLO. The above mentioned study ensure that deep learning framework is an optimal method for pavement inspection task, i.e., crack and garbage detection.

### 1.2. Objectives

Taking account of the above facts, the objectives of present paper fixed as: (a) Incorporating deep learning-based vision system for pavement segmentation on Panthera, (b) Detection of the pavement cracks, (c) Geo tagging of the pavement cracks after detection for effective monitoring, and (d) Deep Convolution Neural Network (DCNN) based vision system for cleanliness inspection task.

The rest of the paper is organized in five sections as follows: Section 2 gives the brief overview of the Panthera robot mechanical, sensory, and electrical components. Section 3 describes the defects and garbage detection framework. Experimental results are discussed in Section 4. Conclusions and future work are finally presented in Section 5.

## 2. Panthera Robot Architecture

**Figure 1:**
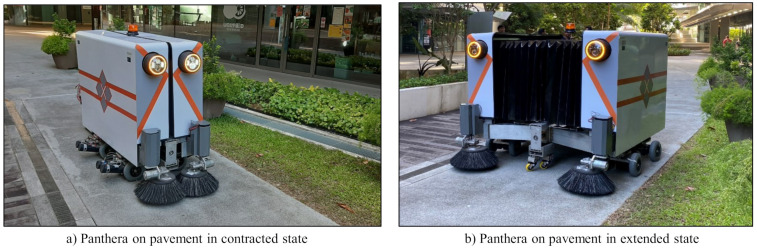
Self-reconfigurable pavement inspection and cleaning robot Panthera.

The design and specifications of the the self-reconfigurable pavement inspection and cleaning robot Panthera are discussed here for brevity. Figure 1 shows the reconfiguration on pavement and the robot specifications are listed Table 1.

The proposed architecture has adopted the following key features include

- Self-reconfigurable pavement sweeping robot Panthera can reconfigure its shape with contracted and extended state shown in Figure 1a,b respectively. This feature enables it to travel on different pavement widths, avoid static obstructions, and response to the pedestrian density.
- With reconfigurable mechanism, Panthera can access variable pavement width.
- Panthera has omnidirectional locomotion, which helps in taking sharp turns, avoiding the defects, potholes, etc.
- The Panthera is equipped with vision sensors to detect the garbage or litters on the pavement and also the cracks present on it using the images taken during daylight conditions.

### 2.1. Mechanical Components

**Figure 2:**
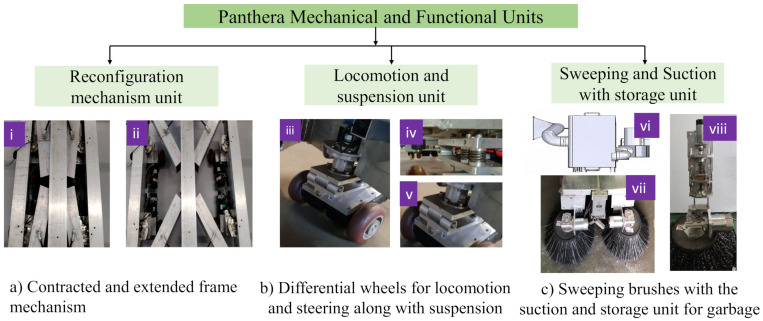
Mechanical and other functional components of Panthera.

Figure 2 shows the mechanical system and key functional components of Panthera robot. It comprises of (a) reconfigurable mechanism unit (b) locomotion and steering mechanism unit and, (c) sweeping and suction unit.

#### 2.1.1. Reconfigurable Mechanism

The reconfiguration unit consists of the expanding and contracting mechanism as shown in Figure 2a. The central beam contains a machined shaft with a single-lead Acme threaded screw. The shaft has right-handed threads in one half and left-handed thread in another. The dimension of the Panthera in its full extended and retracted state are 1.75 × 1.70 × 1.65 meters and 1.75 × 0.80 × 1.65 meters respectively and is shown in Figure 1. The power sources, vacuum units suction drum were accommodated on the central, left, and right beam or frames.

#### 2.1.2. Locomotion and Steering Units

The locomotion and steering action is attained using four steering units having two in-wheel motors in each resulting in the thrust for locomotion provided by eight powered wheels. Each steering units have two in-wheeled motors resulted in a differential wheel, as shown in Figure 2b (iii). The rotation sequence of the eight wheels will result in the steering or locomotion. When all the eight wheels are synchronized to rotate in one direction, then forward or backward locomotion is obtained. For sideways locomotion, the steering units are turned by $90^{\circ }$ first by the rotating the two wheels in each steering unit using differential drive pivot turn. Hence the omnidirectional feature of the platform is achieved. The suspension unit is shown in Figure 2b (iv) which is pivoted to move about an axis as shown in Figure 2b (v).

#### 2.1.3. Sweeping and Vacuum Units

Figure 2c shows the sweeping and vacuum units. The suction motor attached to the collecting box with the inlet opening of the diverging section is shown in Figure 2c (vi). The sweeper brushes, as shown Figure 2c (vii), move the materials from the pavement towards the vacuum cleaner inlet duct with an opening dimension of 20 × 14 cm, which is the height of a typical cool-drinks can. The scrubbing brushes, that are actuated using the rotary motors are made to engage and disengage against the ground by the screw action, as shown in Figure 2c (viii). Note that the identification of the garbage on streets becomes essential for twofold reasons (a) For effective cleaning of dirty areas (b) With the limited dimension of vacuum inlet of these machine objects bigger in dimension should be avoided else it may jam the vacuum inlets.

### 2.2. Electrical and Programming Units

**Figure 3:**
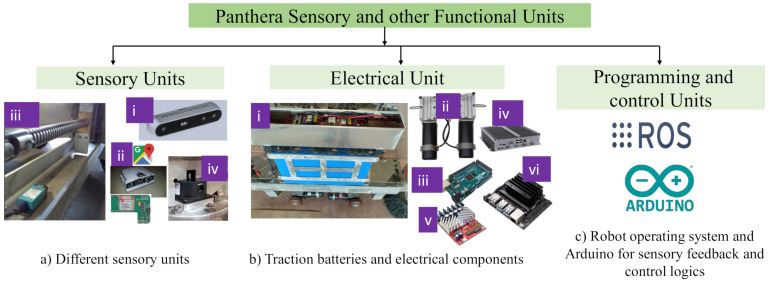
Sensory, electrical, programming and control units in Panthera.

Figure 3 illustrates the electrical and functional components which consists of (a) sensory units, (b) electrical units, and (c) programming and control units.

#### 2.2.1. Sensory Units

Panthera sensory system comprise of various sensing devices include mechanical limit switch, Intel Realsense depth sensor, absolute encoder and GNSS tracking module. A short description for the sensory units are as follows:

- Vision system: Intel Realsense D435 depth camera is utilized in panthera vision sensor as shown in Figure 3a (i). The camera has wide field of view ($85.2^{\circ }\times 58^{\circ }\times 94^{\circ }$) and high pixel resolution 1920 × 1080 which is fixed in center of the front panel of Panthera robot.
- Global Navigation Satellite System (GNSS) receiver was used for getting the geographical location of the defect region, as shown in Figure 3a (ii). The GNSS device namely NovAtel’s PwrPak7D which is robust and accuracy of 2.5 cm to few meters A 16 GB internal storage device is used to log the locations of the robot. The device is shown in Figure 3a (ii). The accuracy can further be improved by combining the wheel odometry data along with the filters.
- Mechanical limit switches: In order to to limit the the reconfiguration of the robot between 0.8 to 1.7 meters the mechanical switch was used Figure 3 a (iii). Limit switch with roller type plunger is attached at the end of the lead screw to limit the movement of the re-configuring frame safely. The limit switch, when triggered, results in an immediate stop in the rotation of the lead screw shaft.
- Absolute encoders: Absolute encoders were used to get the feedback for the steering rotation achieved by differential action of the wheels, as shown in Figure 2b (iii) and Figure 3a (iv). The A2 optical encoder from US Digital was mounted on top of the four steering units and communicated using RS-485 serial bus utilizing US Digital’s Serial Encoder Interface (SEI) was used.

#### 2.2.2. Electrical Units

The electrical unit block with the traction batteries mounted on the frame of the robot is shown in Figure 3b (i). The traction batteries of 24 Volts connected in parallel to power the sweeping robot Panthera. The differential wheels attached with the hub is connected directly to the DC brushed geared motor Figure 3b (ii) of 24 Volts and 130 rpm. To power these actuators, Roboclaw as shown in Figure 3b (v) of 24 and 30 Amperes rating was used to provide electrical pulses as per the control velocity set in logic written for micro-controller board based on the ATmega328P, here Arduino Mega shown in Figure 3b (iii).

#### 2.2.3. Control Units

**Figure 4:**
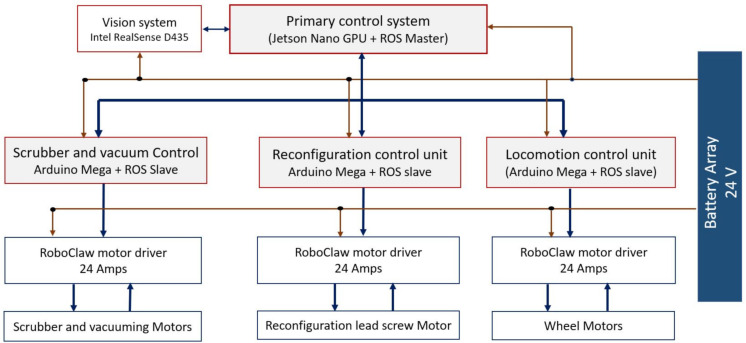
Hardware architecture.

Figure 4 shows the block diagram of the Panthera control system hardware architecture. The hardware architecture comprises of five control blocks includes the primary control system, Deep Neural Network Processing (DNNP) unit, localization control, reconfiguration control, and cleaning module control unit. The primary control system is built with an industrial PC, which comprises 8 core CPU,16GB RAM, and separate GPU cards for running deep neural network function in real-time. Here, the primary system uses GPU for running the SegNet framework and NVIDIA Jetson nano GPU embedded board for run the inspection CNN module. The NVIDIA Jetson nano comprises of ARM A57 CPU and 128 Core maxwell GPU with 4GB memory and running on Ubuntu 18. The primary control system contains the Robot Operating System (ROS) master function, which generates the control message to the Jetson nano GPU, localization, reconfiguration, and cleaning module. The central processing unit utilizes a server-client communication model to communicate with other modules. The real sense vision module is connected to the primary control system unit using a USB 3.0 communication interface. The TensorFlow an open-source deep learning framework is configured in both units to run the deep learning function.

The other control modules include localization, reconfiguration, and cleaning device control unit are powered with Arduino-Mega microcontroller and configured as ROS slave for communicating with the primary control system. The slave units handle the various sensor interface and generate the required control and Pulse Width Modulation (PWM) signal to motor drivers.

## 3. Defect and Garbage Detection Framework

**Figure 5:**
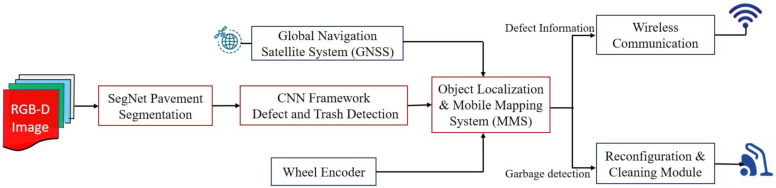
Pavement defects and cleanness inspection framework.

Functional block diagram of pavement inspection framework is shown in Figure 5. It comprise of pavement segmentation module, defect and garbage detection and defect localization module.

### 3.1. Input Layer

The input layer takes in the raw image obtained after sampling the video into images. The input layer converts each frame into a specific size of the image. Here, input layer that resizes the extracted images into 640×480 pixels.

### 3.2. Pavement Segmentation

**Figure 6:**
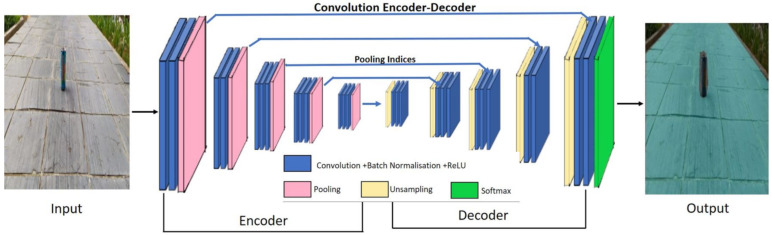
SegNet block diagram.

Figure 6 shows the SegNet [37] based pavement segmentation module. SegNet is an Deep Learning (DL) based Semantic image segmentation framework which is widely used in autonomous driving vehicle, industrial inspection, medical imaging, and satellite image analysis. In this work, SegNet DCNN module was adopt to segment the pavement region from other objects.

The SegNet architecture is comprised of deep convolution based encoder layer and a corresponding set of decoder layers followed by using a pixel-wise classification layer. The encoder and decoder part consists of thirteen convolution layers, Rectified Linear Unit (ReLU) activation function, and 7×7 kernels based max-pooling layers. At the encoder side, convolution and max-pooling operation are performed. Similarly, up-sampling and convolutions operation is executed at the decoder side. While performing max-pooling operation in the encoder side, corresponding max-pooling indices (locations) are stored for use in decoding operation. Finally, K- class softmax classifier is connected with decoder output to compute the class probabilities for every pixel individually. For retaining the higher resolution feature maps, fully connected layers are removed at deep encoder output. Furthermore, a Stochastic Gradient Descent(SGD) algorithm is used for train and optimize the SegNet framework with a learning rate and momentum of 0.002 and 0.9, respectively. A training set with 20 samples is used for training CNN. The model with the best performance on the validation data set in each epoch was selected.

#### 3.2.1. Pavement Defects and Garbage Detection

**Figure 7:**
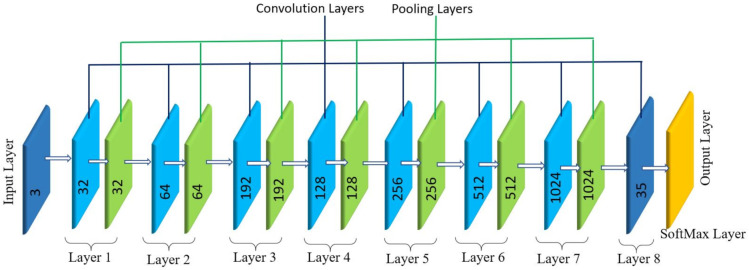
CNN architecture and layers description.

Multi-layer CNN model for defect and garbage detection task is shown in Figure 7. Here, Dark flow framework has been used to build the multi-layer CNN model which is an python-based network builder with a TensorFlow backend. The difference between a Dark flow and traditional CNN is that the in traditional CNN, the classifier uses an entire image to perform any classification whereas in a Dark Flow based network, the images are split into multiple grids and within each grid for generating multiple bounding boxes. A trained model outputs the probability that an object is present in a bounding box and if that probability goes above a specified value in a specific bounding box, then the algorithm extracts features from the that specific part of the image to locate the object.

In short, in a dark flow-based network, the network searches for the desired objects in regions that have higher probability than the threshold value as opposed to searching it throughout the entire image which more exhaustive and prone to error. This makes it a much cleverer CNN for performing object classification and localization and it is faster than many other model builders as it only carries out prediction in selected grid cells which makes it a great candidate for using it in real-time.

The multi-layer CNN (Figure 7) consist of 9 convolutional layers, 6 pooling layers and an output layer with softMax activation function. The number below the curved braces gives the information of all the layers such as padding, and the number inside, i.e., 32,64,192, etc., represents filter size and stride.

#### 3.2.2. Convolutional Layers

Convolutional layer repeatedly performs the operation of convolution between the input image and chosen filter. The operation of convolution involves performing a element-by-element multiplication of a sub array of input image with the chosen filter and the result of each of these element-by-element multiplication is summed which corresponds to an one single element in the output of the convolutional layer. The size of the sub array is equal to the size of the filter. Once the result is obtained from a sub array of the input image, a different sub-array is chosen to perform the similar operation. The new sub-array is selected based on the stride length (s) i.e., the new sub-array would be chosen by shifting across a specified number of rows and columns from the previous sub-array and this convolution operation is continued until the entire input image is covered. There are various hyperparameters involved in this operation: stride length(*s*), padding (*p*), filter size (*K*). The stride length determines the number of units that will be shifted by the filter at one time. In other words, the stride is the amount by which the filter shifts to perform the convolution. The padding determines the number of zero padding layers applied to the input data before performing the convolution operation. In this work, valid padding was used with null zero padding (p=0). The operation of the convolution has been represented by the following Equation (1):

(1) $$\left(f*g\right)\left(t\right)≜\int_{-\infty }^{\infty }f\left(\tau \right)\;g\left(t-\tau \right)d\tau$$

where, *f* donates input function and *g* denotes filter function of convolution.

#### 3.2.3. Pooling Layer

The general rule of thumb in constructing a CNN network involves a convolutional layer followed by a pooling layer. This is done to enhance the feature extraction process while reducing the spatial dimension of the input from the preceding layer. This process of reducing the spatial dimension is known as down sampling and this process improves the efficiency and speed of the network. Moreover, it is used to generalize the model by reducing overfitting. The pooling layer applies non-linear down sampling on the activation maps which, in turn, reduces the spatial size of the representation. This layer also reduces computation time by reducing the number of parameters required. In this paper, Max pooling was used as a filter for this layer. In max pooling layer, the maximum value within the region covered by the filter is taken and assigned as the output value.

#### 3.2.4. SoftMax Layer

The final layer in the proposed network is the SoftMax layer. SoftMax layer is usually for classification as it provides a probabilistic distribution of the classes. The class that has the highest probability is provided as the result. This can also be looked at as an activation function.

#### 3.2.5. Activation Function

In the proposed network, leaky Rectified Linear Unit (ReLU) is used as the activation function for the convolutional layers and the linear for the final convolutional layer. The need for non-linearity in the network requires us to pick leaky ReLU as the activation function. The Equations (2) and (3) indicates the underlying mathematical operation corresponding to Leaky ReLU.

(2) f(z)=az,ifz<0

where a = 0.01

(3) f(z)=z,ifz>0

The Equation (4) indicates the mathematical operation for linear function.

(4) f(z)=w∗z

#### 3.2.6. Bounding Box

This work is focused on object localization. Customized CNN network is used to find the Region of Interest (RoI) or exact location in the color image. The output from the above process is wrapped inside bounding box. The bounding box is constructed to divide the images into segments of equal area and to generate target vector for them using Equation (5). The bounding box for training would be as follows.

(5) $$y={\left[P\;X_{min}\;Y_{min}\;X_{max}\;Y_{max}\;C_{1}\;C_{2}\right]}^{T}$$

where *P* is the binary value which determine the object of interest in the image, $X_{min}$ and $Y_{min}$ is upper left *X* and *Y*-coordinate of the bounding box. Similarly, $X_{max}$ and $Y_{max}$ lower right *X* and *Y*-coordinate of the bounding box. The value of the argument $C_{1}$ will be one if belongs to class 1 else zero. Similarly, the value of $C_{2}$ is equal to one if the object belongs to class 2, else zero. Furthermore, Intersection Over Union (IOU) method Equation (6) is utilized to eliminate the overlapping bounding boxes. IOU is the ratio of area of overlap to the area of union.

(6) $$IOU=\frac{\left(A\cap B\right)}{\left(A\cup B\right)}$$

In this work, IOU threshold is fixed to 0.5. If the calculated IOU and actual IOU of bounding box is equal to or more than 0.5, then the obtained output is correct, else the it will be considered as false prediction by bounding box coordinates. Table 2 shows the average IOU matching of all bounding boxes in text set.

### 3.3. Mobile Mapping System for Defect Localization

**Figure 8:**
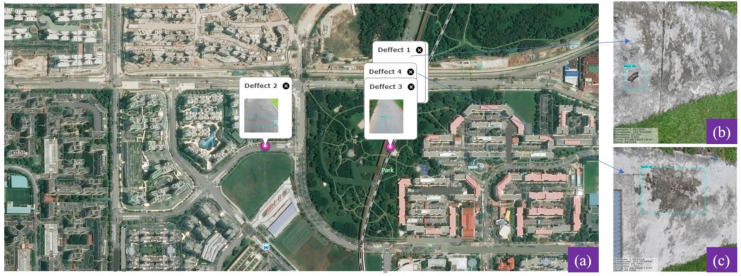
Independent trial for the mobile mapping system and the defect identification. (**a**) Defect location on global map, (**b**,**c**) detected defects and patches.

Mobile Mapping System (MMS) [38,39] is the final component of proposed system, which is used for tracking the location of the defects. The MMS technique stamped the Geo referencing parameters into defect detected images and forward to a remote monitoring unit or PMS for finding and fixing a pothole or other defects. The MMS function was run in the primary control unit and used three data for accomplishing the defect localization includes physical distance data *d* estimated by realsense rs-measure API function, and two hardware modules data such as wheel encoder which provide the odometeric distances and Global Navigation Satellite System (GNNS) data which provide latitude and longitude information. The generated MMS data is forward to a remote monitoring unit through a 4G wireless communication module. Figure 8 shows one of the independent test trials, i.e., carried without the Panthera robot being moved in the public park connectors. The image highlights the location of the defects on the map which will assist in the faster maintenance of the pavement.

## 4. Experiments and Results

This section describe the experimental results of proposed system. The experiment has been performed in three phases: data set preparation, training the SegNet and inspection CNN (defects and garbage detection), and validating the trained models. Generally, the detection or segmentation performance of DNN relies on various parameters, including the size of the training data set, data-set class balance, illumination conditions, and hyper-parameters (learning rate, batch size, momentum, and weight decay). These parameters play a key role in network performance and computational cost in both training and testing phases. Learning rate directly affects the time taken for the training to converge. A small learning rate makes the training longer, whereas a high learning rate may lead to large weight updates and overshoots. The batch/sample size affects the computational cost and should be chosen according to hardware memory. Hence, these parameters need to be tuned appropriately. After some trials, it is found that a learning rate of 0.02, a momentum of 0.9, and a training set of 20 samples provide satisfactory results in our application.

### 4.1. Data set Preparation and Training the Model

**Figure 9:**
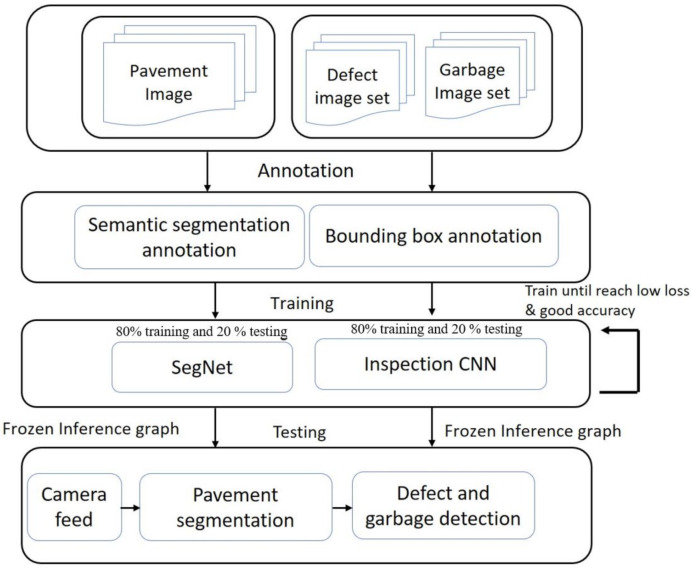
Flow diagram for training the pavement inspection framework.

The dataset preparation, training, and testing flow diagram for proposed system as shown in Figure 9.

Here, the data-set preparation process involves collecting the pavement images, pavement defects, and garbage images with different pavement background. The data-set consists of 3000 image samples of pavement collected from different locations in Singapore. The image acquisition is done from the robot perspective under different lighting conditions. The collected images are balanced for two different classes (1) pavement defect (cracks and damages) and (2) garbage (tissue paper, food packing paper, polythene cover, metal bottle, and plastic).

For training and testing the model, fixed resolution of the image size 640×480 was used throughout the experimental trials. In-order to enhance the CNN learning rate and to control the over-fitting, data expansion techniques such as rotation, scaling, and image flipping were used in the training phase. The K-fold cross-validation process is utilized for the model assessment (In this case K = 10 was fixed). The data-set is divided into 10 sections and one among the 10 sections is used for testing the model and the remaining 9 has been used for model training. To eliminate biasing conditions due to a particular training or testing data-set, this process has been repeated 10 times. Besides, the results from the performance matrix are repeated over 10 times and the mean results are provided. The resulting images from the highest accuracy models are given here.

The CNN models SegNet and inspection are realized using Tensor-flow 1.9 module on Ubuntu 18.04 operating system. The models are trained using a computer that uses Intel Core i7-8700k, 64 GB of RAM, and NVIDIA GeForce GTX 1080 Ti Graphics Card. To assess the performance of the proposed scheme, standard statistical methods such as accuracy, precision, and recall was adopted. Equations (7)–(10) shows the accuracy, precision, recall and $F_{measure}$ respectively.

(7) $$Accuracy\left(Acc\right)=\frac{tp+tn}{tp+fp+tn+fn}$$

(8) $$Precision\left(Prec\right)=\frac{tp}{tp+fp}$$

(9) $$Recall\left(Rec\right)=\frac{tp}{tp+fn}$$

(10) $$F_{measure}\left(F_{1}\right)=\frac{2\times precision\times recall}{precision+recall}$$

(11) $$\eta_{miss}=\frac{\eta_{missnum}}{\eta_{testset}}\times 100\%$$

(12) $$\eta_{false}=\frac{\eta_{falsenum}}{\eta_{testset}}\times 100\%$$

As per the standard confusion matrix, the variables tp,fp,tn and fn are true positives, false positives, true negatives and false negatives respectively. The $\eta_{miss}$ represents the target object not recognized by the network and $\eta_{false}$ represents objects detected as target object.

### 4.2. Validation of Defect and Garbage Detection Framework

**Figure 10:**
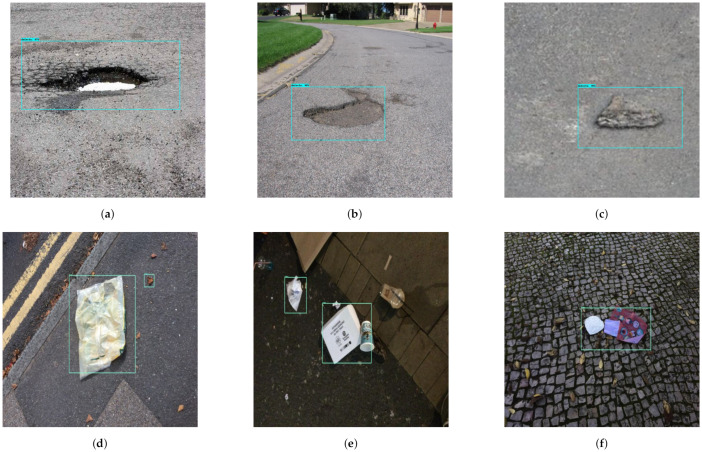
Offline test results for defect detection (**a**–**c**) and garbage detection (**d**–**f**).

After training the model, the effectiveness of the trained CNN framework was tested in offline and real time mode with Panthera. To carry out the offline experiments, the trained model was loaded into Jetson nano and tested in laboratory with locally collected 1200 defect and garbage’s images. Figure 10 shows the detection results of defect and garbage’s tested in offline. In this experiment the detection model detect most of defect and garbage with higher confident level with mean average precision (mAP) of 92% for defect and 95% for garbage.

In order to evaluate the real time defect and cleanness inspection, six hundred meter pavement was selected as a test bed which is located near our institution. Before carrying out the experiment, the pavement defect region are manually notified which is used to compare the detection results of proposed system. For cleanness inspection, different type of garbage are randomly drop on the test bed and its detection was tested by model.

**Figure 11:**
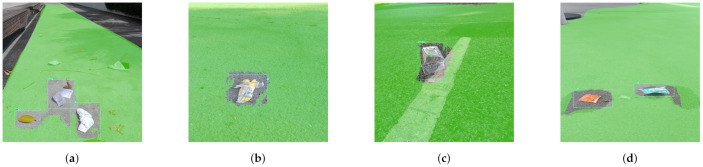
Pavement segmentation and the garbage detection results in (**a**–**d**).

**Figure 12:**
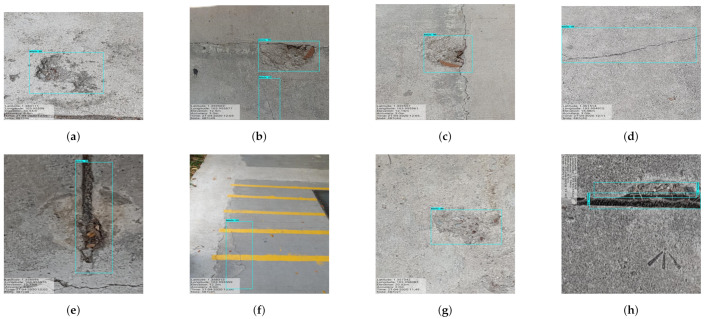
Defects with geotagging information in (**a**–**h**).

**Figure 13:**
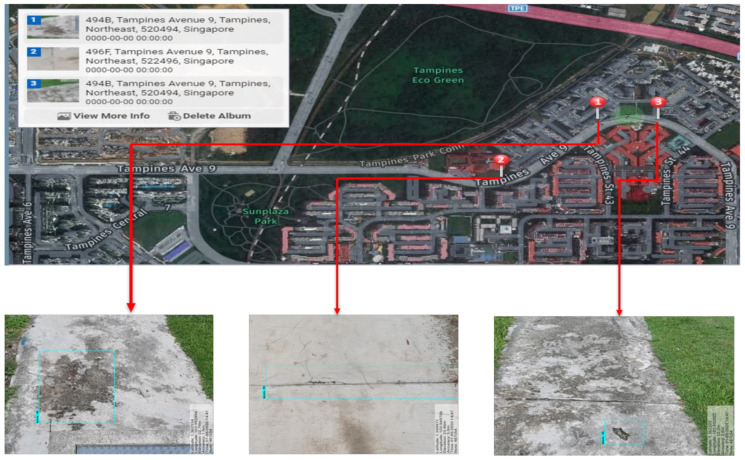
Defect location mapped on Google map.

**Figure 14:**
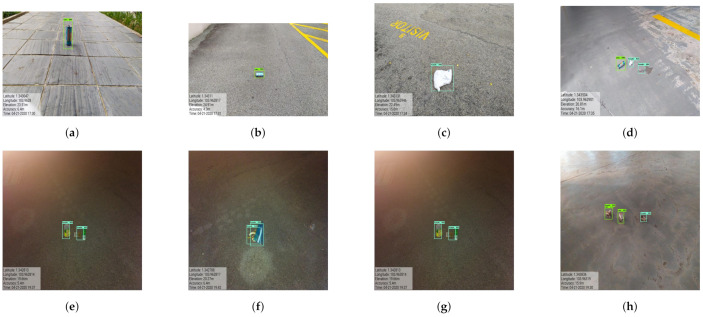
Pavement garbage detection in real-time in (**a**–**h**).

In this experiment, the images are capture from the Panthera robot operating inside the university campus pavement. The vision module runs at 10 frames per second (fps), and image resolution was set to 640×480. The robot was moved at a slow speed on the pavement, and the detection results are recorded from a remote monitoring unit. Figure 11 shows the segmentation and real-time detection of the garbage on the pavement. Figure 12 and Figure 13 shows the defects detected on pavement along with geotagging and google mapped results. Furthermore, Figure 14 shows the garbage detection resulst of inspection framework along with their Geotagging information. The defects on the pavement detected are marked by sky-red rectangle box and garbage detected are marked by green rectangle box. In this analysis, the detection model detects a defect with the mean Average Precision (mAP) of 88% to 92% confident level and garbage with 91% to 96% confidence level, respectively on the data set used.

Furthermore, statistical measures has been performed for estimating the robustness of the detection model for both online and offline experiments. Table 3 shows the statistical measures result for online and offline experiments.

The statistical measure indicate that the trained CNN model has detect the defect with 93% precision for offline test and 89.5% precision for online test. Furthermore, the model miss rate is 2% for offline test and vary 4% to 7% for online test due to different lighting conditions. Similarly, the garbage’s are detected at precision of 96% for offline test and 91.5% for online test. However its miss detection ratio is quite low compare to defect detection for different lighting conditions. It is due to higher visibility of garbage compare to defects. The study ensure that the proposed system was not heavily affect by environment factor like varying lighting condition and shadows. It was ensured by miss detection metrics. In this study, miss detection ratio difference is less than 4% for environmental changes. However, 4% of miss detection are due to the invisible defects or the defects region have been heavily covered by shadows.

Further computational cost of the models was tested by time taken for processing one 640×480 resolution image frame on execution hardware. Here, SegNet model was executed on primary control system and detection model was executed on Jetson Nano embedded GPU board. The experiments was tested for 100 images and its detection times are recorded. The experimental results shows that the SegNet model takes average of 120 ms to segment out the pavement region from 640×480 image frame. Furthermore, the detection model and MMS function took average 12.2 ms for detect the defect and garbage’s. The experiment results shows that the trained model process seven to eight frames per second in average.

### 4.3. Performance Comparison with Other Semantic and Object Detection Framework

Table 4 and Table 5 shows the performance comparison of proposed frameworks for semantic segmentation and object detection framework with other popular other framework. FCN-8 and UNet framework are consider for semantic segmentation model comparison analyses. Similarly, Faster RCNN ResNet, SSD MobileNet are taken as object detection models for comparison. All models are trained using the same dataset consisting of 3000 images and similar training time on the same GPU card. The comparison has been detailed in Table 4 and Table 5. The performance of comparison is made based on standard performance comparison matrices. The experimental analysis indicate that SSD-MobileNet and proposed system have comparable accuracy of 94.64% and 95.00%. However, in terms of execution time and detection accuracy, Faster RCNN ResNet, has the upper hand over SSD-MobilNet and proposed system. Here, the trade-off between the models’ computational expense and accuracy are key parameter for chosen dark flow based proposed system as the candidate for trash detection task, considering the possibilities of enhancing the detection accuracy of proposed system with a lower training rate. Similarly, performance comparison of segmentation frameworks SegNet yields better pixel-wise classification than other networks. Besides, the accuracy of FCN8 and UNet drops significantly when it comes to the object classes with small pixel areas.

#### Validation with Other Defects and Garbage Image Database

The effectiveness of defect and cleanness inspection model was tested with Cui et al crack forest [40], Fan Yang pavement crack [41], Hiroya Maeda road damage [42], taco [43], and Mindy yang trashnet [33] image data sets. The defect image databases [41,42,44] contains annotated pavement and road crack images captured at different pavement and urban road surfaces. The taco and Mindy yang image data set contains various kinds of paper trash, plastic and metal cans taken under diverse environments.

**Figure 15:**
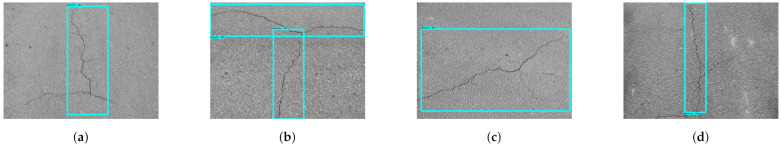
Defect detection results for Fan Yang cracks dataset in (**a**–**d**).

**Figure 16:**
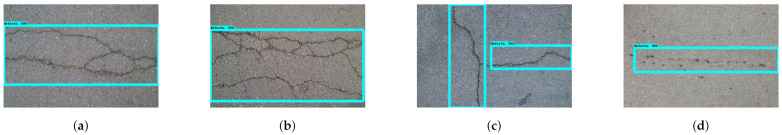
Defect detection results for CrackForest dataset in (**a**–**d**).

**Figure 17:**
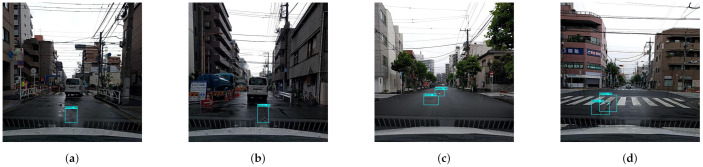
Defect detection results for Hiroya Maeda road damage data-set in (**a**–**d**).

**Figure 18:**
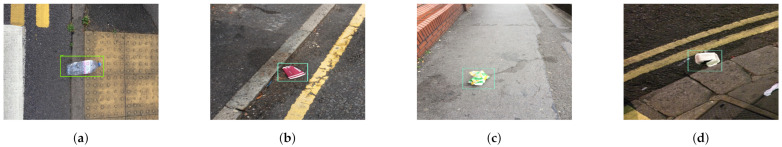
Garbage detection results for Taco trash image dataset in (**a**–**d**).

**Figure 19:**
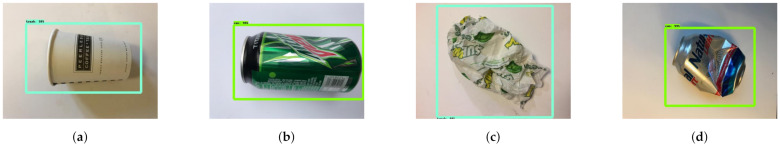
Garbage detection results for Gary Thung and Mindy Yang Trashnet dataset in (**a**–**d**).

The experiment results for defect and garbage image database are shown in Figure 15, Figure 16, Figure 17, Figure 18 and Figure 19 and its statistical measure are reported in Table 3. Over 150 images are taken from each of the database for perform the statistical analysis.

The statistical results, given in Table 6 shows an average of 94.6% confidence level for detecting the defects and average of 95.5% confidence level for detecting the garbage.

### 4.4. Comparison with Existing Schemes

This section describes the comparative analysis of the proposed system with existing pavement defect and garbage detection case studies in the literature.

The comparison has been made based on a deep learning framework used for both pavement defects and garbage (dirt, garbage, marine debris) detection task. The detection result of various defect and garbage detection schemes are shown in Table 7 and Table 8. The difference analysis has been reported based on CNN topology and detection accuracy.

In this comparison analysis, we try to provide some fair comparison with proposed system and existing scheme based on some key differences. From above table, Crack U-net [50] and Deep encoder-decoder [25] are based on pixel-wise crack detection architecture and trained with 3000 and 600 defect image respectively. Here, Crack U-net obtained detection accuracy of 99.01%, which follows the FCN style CNN layers. Similarly, the deep encoder-decoder framework was constructed with a residual network convolution layer and gained quite lower precision 59.65% than Crack U-net. However, both models need a larger computing resource and less suitable for in-situ inspection. In [25,48] implementations are developed for real-time remote defect inspection where multi-layer CNN and drone are used by Naddaf-Sh et al. and model process 5 frames per second and achieve 96.00% detection accuracy [25]. other-hand FCN - Gaussian-conditional random field combination was used by Zheng Tong et al.. The model was tested with high computing device NVIDIA GeForce GTX 1080 GPU fitted on multi-function testing vehicle and takes 0.162 inference time and obtained a precision of 82.20% [48].

Further other implementation such as YOLO v2 [29,49], SSD MobileNet [42], SSD inception [42] and CrackNet [50] which are light-weight framework. Its architecture is more suitable for real-time on-site inspection and also runs in low power computing devices. However those models are used only on offline defect inspection and obtained detection accuracy of 88.00% [29], 93.00% [49] 77.0% [42], 99.00% [50] respectively.

In contrast with the above implementation, the proposed model work in real-time pavement defect detection, and its average detection accuracy is 93.3%. The proposed CNN model was constructed with low number of convolution layers (16), which help to reduced computational demand and results in real-time processing. The number of the hidden layer has also reduced after convolution and max-pooling layers. In addition to that, the training data in each class made equivalent to training to avoid data skew. Furthermore, MMS based localized defects have an added advantage for the proposed framework and help in the pavement monitoring system (PMS).

Table 8 shows the overview of CNN based garbage detection which are developed various garbage cleaning application. Here, SegNet and ResNet combination [51] was developed for garden cleaning robot for detecting garbage on grass and obtain 96% detection accuracy. Mobilenet V2 SSD was used in floor trash detection and achieve 95.0% detection accuracy. However that implementation not tested in any cleaning robot. The other two implementation are based on marine garbage detection using two different framework such as Faster RCNN InceptionV2 and MobileNetV2 with SSD. Here, under water vehicle was used for capture the marine garbage and tested in offline and models obtained moderate detection accuracy 81.0% and 69.0% respectively. In contrast with above mention scheme, the proposed model detect the garbage with an average of 95% detection accuracy.

The preset framework is limited to the inspection task of pavements during the day light conditions. The detection model is limited to very few objects, namely tin can, papers, and plastic bottles. Also the inspection is limited to the crack detection which can not differentiate between pothole, bumps, etc. Also the speed at which the detection task was carried out by the robot is limited to 0.1 m/s and with a vision feedback decimated at a rate of 10 frame per seconds. Further cleaning module has some generic limitations, including it cannot pick up garbage bigger than the dimension of the vacuum inlet opening dimensions as indicated in the section “Sweeping and vacuum units.” Furthermore, it is unable to clean up heavy objects which are not classified as garbage. Some heavy objects which are small might be stones. The weight that the vacuum can carry depends on the vacuum motor power. However, it is noted that vacuum motor power can be changed easily by changing the motor.

## 5. Conclusions and Future Work

In this article, the pavement defect and cleanness inspection using a deep learning based framework was proposed and implemented in the self-reconfigurable pavement sweeping robot Panthera. A lightweight DCNN model was developed and trained with 6000 defect and garbage images. The framework was configured in Jetson Nano NVIDIA GPU and took approximately 132.2 milliseconds for detecting both pavement defects and garbage. Moreover, the geotagging of the pavement defects was presented during locomotion on the pavement. The experimental results show that the proposed method identifies the pavement defects and garbage with 93.3% and 95.0% detection accuracy, respectively. The framework is an initial attempt to use a DCNN framework on pavement cleaning robots for inspection, which includes defect and garbage detection tasks. The application of this work is in a pavement management system where the existing sweeping vehicle can use its sensory feedback from the vision system for the inspection task using a machine learning framework.

Our ongoing efforts focus on implementing a depth sensor feedback in the pavement monitoring system for further classification of localization of garbage and crack detection. Also, the enhance detection and segmentation framework by train the network rich data sets taken under different lighting conditions are targeted. Furthermore, the scheme for crossing or avoiding the cracks, collecting or not collecting the detected garbage, are being developed for Panthera. Also the framework to scaled for the identifying defects in drains using drain inspection robot.

## Figures and Tables

**Table 1:** Panthera specifications.

Parameter	Dimension	Unit
Panthera height	1.65	m
Panthera width (Retracted)	0.80	m
Panthera width (Extended)	1.70	m
Side brush’s diameter	0.27	m
Wheels radius and numbers	0.2, 8	m, unit
Drive	Differential	–
Driving power	700	W
Turning radius	Zero	m
Continuous working time	>4	hrs
Working speed	3	km/h
Driving speed	5	km/h
Net weight	530	Kgs
Payload	150–200	Kgs
Platform locomotion	Omnidirectional	–
Power source	Traction batteries DC	24 V
Sensors	RGBD Camera	–

**Table 2:** Evaluation metrics associated to the bounding box predictions.

Average IOU Matching	Over All Confidence
0.7044	0.589

**Table 3:** Statistical measures for defect and garbage’s detection.

Test	Offline Test			Online Test			Other Metrics		
	Precision	Recall	$F_{1}$	Precision	Recall	$F_{1}$	$\eta_{\mathrm{miss}}$	$\eta_{\mathrm{false}}$	Overall Accuracy
Defect	93.0	91.8	92.1	89.5	87.1	86.67	4	2	93.3
Garbage	96.0	94.43	93.22	91.5	87.55	87.0	7	3	95.0

**Table 4:** performance comparison of segmentation frameworks.

Semantic Framework	Pixel-Accuracy	IOU	F1 Score
FCN-8	85.33	86.72	86.89
U-Net	88.12	88.56	88.18
SegNet	93.30	93.18	90.93

**Table 5:** performance comparison of object detection frameworks.

Semantic Framework	Accuracy	Precision	Recall
Faster RCNN ResNet	97.89	96.30	96.82
SSD MobileNet	94.64	93.25	92.88
Proposed system	95.00	93.11	92.66

**Table 6:** Case study for the Convolution Neural Network for road defects.

Database	Precision	Recall	F1	Average Confidence Level
Crack forest (Defect) [40,44]	94.5	91.8	92.2	96.0
Fan Yang (pavement crack) [41,45]	93.3	89.7	90.0	95.0
Road Damage Dataset [42]	92.3	88.5	89.0	93.0
TACO [43]	96.0	93.2	92.5	97.0
Trashnet [33]	94	98.5	97.6	94.0

**Table 7:** Comparison with other detection schemes.

Case Study	Inspection Type	Algorithm	Detection Accuracy
Ju Huyan et al. [46]	Offline	CrackU-net	98.56
Bang et al. [47]	Offline	deep encoder-decoder network	90.67
Naddaf-Sh et al. [25]	Real time with drone	multi layer CNN	96.00
Zheng Tong et al. [48]	Multifunction testing vehicle	FCN with a G-CRF	82.20
Mandal et al. [29]	Offline	YOLO v2	88.00
Majidifard et al. [49]	Offline	YOLO v2	93.00
Maeda et al. [42]	Real time with smartphone	SSD MobileNet	77.00
Zhang et al. [50]	Offline	CrackNet	90.00
Proposed system	Online and offine	16 layer CNN	93.30

**Table 8:** Comparison with other garbage detection schemes.

Case Study	Algorithm	Detection Accuracy
Garbage detection on grass [51]	SegNet + ResNet	96.00
Floor trash detection [35]	Mobilenet V2 SSD	95.00
Garbage detection on marine [52]	Faster RCNN Inception v2	8100
Garbage detection on marine [52]	MobileNet v2 with SSD	69.00
Proposed system	16 layer CNN	95.00

## Data Availability

Not applicable.

## Abbreviations

Lists the abbreviations and nomenclature used in this paper.

Term	Explanation
GPU	Graphical Processing Unit
CNN	Convolutional Neural Network
DCNN	Deep Convolutional Neural Network
MMS	Mobile Mapping System
PMS	Pavement Management System
ML	Machine Learning
DL	Deep Learning
VGG16	Visual Geometry Group 16
YOLOV2	You Only Look Once
CAR	Classification Accuracy Rate
F-RCNN	Fast Region Convolution Neural Network
SVM	Support Vector Model
SIFT	Scale Invariant Feature Transform
SSD	Single Shot Detector
GNSS	Global Navigation Satellite System
GB	Giga Byte
SEI	Serial Encoder Interface
DNNP	Deep Neural Network Processing
RAM	Random Access Memory
ROS	Robot Operating System
PWM	Pulse Width Modulation
DL	Deep Learning
ReLU	Rectified Linear Unit
RoI	Region of Interest
IOU	Intersection Over Union
4G	Fourth Generation
tp	true positives
fp	false positives
tn	true negatives
fn	false negatives
Acc	Accuracy
Prec	Precision
Rec	Recall
F1	F1measure
fps	frames per second
MAP	Mean Average Precision
FCN-8	Fully Convolutional Neural Network
SGD	Stochastic Gradient Descent
$X_{min}$	upper left *X* coordinate of the bounding box
$Y_{min}$	upper left *X* coordinate of the bounding box
$X_{max}$	lower right *X* coordinate of the bounding box
$Y_{max}$	lower right *X* coordinate of the bounding box
